# Change in serum sodium level predicts clinical manifestations of transurethral resection syndrome: a retrospective review

**DOI:** 10.1186/s12871-015-0030-z

**Published:** 2015-04-16

**Authors:** Junichi Ishio, Junko Nakahira, Toshiyuki Sawai, Teruo Inamoto, Atsushi Fujiwara, Toshiaki Minami

**Affiliations:** 1 Department of Anesthesiology, Osaka Medical College, 2-7 Daigaku-machi, Takatsuki, Osaka 569-8686 Japan; 2 Department of Urology, Osaka Medical College, 2-7 Daigaku-machi, Takatsuki, Osaka 569-8686 Japan

**Keywords:** Transurethral resection of prostate, Transurethral resection syndrome, Dilutional hyponatremia

## Abstract

**Background:**

Patients undergoing transurethral resection (TUR) of the prostate are at risk of TUR syndrome, generally defined as having cardiovascular and/or neurological manifestations, along with serum sodium concentrations less than or equal to 125 mmol/l. As these symptoms can also occur in patients with serum sodium greater than 125 mmol/l, this study aimed to investigate the relationship between serum sodium concentrations and neurological manifestations of TUR syndrome.

**Methods:**

Data on patients who underwent TUR of the prostate under local anesthesia over an 8-year period were retrospectively reviewed. Based on their cardiovascular and neurological manifestations, patients were divided into two groups: a symptomatic and an asymptomatic group. Logistic regression analysis was used to detect the risk factors for being symptomatic. Receiver operator characteristic (ROC) curve analysis was used to determine the optimal cutoff value of estimated change in serum sodium level that could predict the development of clinical manifestation of TUR syndrome.

**Results:**

Of the 229 patients, 60 showed symptoms. Serum sodium level correlated with neurological score (Spearman’s correlation coefficient > 0.5). Logistic regression detected that the risk factors for being symptomatic were serum sodium level variables, operation time longer than or equal 90 min, and presence of continuous drainage from the bladder. ROC curve analysis showed that a change in serum sodium level of 7.4 mmol/l was the optimal cutoff value, with a sensitivity of 0.72, a specificity of 0.87, and an area under the curve (AUC) of 0.87. ROC curve analysis also showed that a 7.0% change in serum sodium level was optimal for this parameter, with a sensitivity of 0.70, a specificity of 0.89, and an AUC of 0.87.

**Conclusions:**

Changes in serum sodium concentration of > 7 mmol/l and of > 7% could predict the development of cardiovascular and neurological manifestations, which were assumed to be symptoms of TUR syndrome.

## Background

The incidence of prostate gland enlargement increases with age, occurring in an estimated 50% of males at age 60 years and an estimated 90% at 85 years [1]. Prostatic ultrasonography is used to assess the weight, shape, and structure of the prostate gland and to determine optimal treatment. Transurethral resection of the prostate (TURP) is a standard surgical method used to treat patients with prostate gland enlargement. Our use of monopolar electrodes as electrical cautery for excision increases the necessity of using nonconductive irrigation fluid. These fluids, however, contain no electrolytes, allowing their absorption into the blood stream. This may result in dilutional hyponatremia, which may result in cardiovascular and/or neurological manifestations. In general, transurethral resection (TUR) syndrome is defined as a serum sodium concentration ≤ 125 mmol/l combined with clinical cardiovascular and neurological manifestations [2,3]. Based on this definition, the rate of TUR syndrome has been found to range between 0.5% and 10.5% [2-4]. Experientially, however, symptoms of TUR syndrome may occur in patients with serum sodium levels > 125 mmol/l.

Theoretical risk factors for TUR syndrome include surgical release of the prostatic sinus, high irrigation pressure, prolonged operation time, and use of hypotonic perfusate [2,5]. Moreover, the incidence of TUR syndrome may be significantly higher if surgery takes longer than 90 minutes, if the prostate gland weight is over 45 g, or if patients have acute dysuria, are over 80 years old, or of African race [2,5]. This study investigated whether intra- and post-operative changes in serum sodium concentrations could predict the clinical manifestations of TUR syndrome.

## Methods

Records of patients who underwent TURP under local anesthesia at our hospital from 2006 to 2013 were retrospectively reviewed. The study protocol was approved by the Ethics Committee of Osaka Medical College (reference number 898), which waived the requirement for informed consent due to the retrospective design of the study.

Preoperative spinal anesthesia was achieved via administration of 0.5% bupivacaine hydrochloride (2.0–3.0 ml) at L3/4 or L4/5, resulting in analgesia up to thoracic level 10 (T10). If spinal anesthesia was ineffective, the patient was removed from the study. If the anesthetized region was lower than the T10 sensory levels or operation time was longer than 1.5 h, 0.375% ropivacaine hydrochloride (3.0–5.0 ml) was administrated as epidural anesthesia through a catheter at L1/2 or L2/3, followed by continuous administration of 0.2% ropivacaine hydrochloride (2–5 ml/h) through the postoperative period. Patients with preoperative urethral bleeding disorders, renal insufficiency, or contraindications to spinal anesthesia were excluded.

Irrigation bags filled with 3% D-sorbitol, a non-conductive irrigation fluid for surgery, were fixed at a height of 90 cm above the operating table. Heart rate, electrocardiogram, and percutaneous oxygen saturation were measured and monitored continuously. Systolic blood pressure (SBP) and diastolic blood pressure (DBP) were measured every 2 min. All patients were administered Ringer’s solution, except for those requiring transfusions.

Cardiovascular and neurological manifestations of TUR syndrome were assessed as described by Hahn et al. [6]. As shown in Table 1, cardiovascular manifestations included chest pain, bradycardia, and hypertension. Neurological manifestations included blurred vision, nausea, vomiting, uneasiness, confusion, tiredness, consciousness, and headache. In the patients of this study, we detected nausea, vomiting, restlessness, confusion, abdominal discomfort, and abdominal pain. As blurred vision, consciousness, and headache were difficult to assess, uneasiness and tiredness were included under the category of restlessness. Patients with a > 30% increase in blood pressure relative to preoperative levels measured just before local anesthesia was performed in the operating room, SBP < 80 mmHg, bradycardia, or arrhythmia were immediately treated to avoid further deterioration. Patients with SBP < 80 mmHg were immediately administered 4 mg of ephedrine hydrochloride intravenously. The medical and nursing staff closely monitored patients during and after the procedure to detect and treat complications. Intraoperative anesthetic charts and postoperative nursing charts contained detailed records of each patient’s status. Manifestations of TUR syndrome were differentiated from manifestations of vasovagal reflux caused by filling of the bladder or by spinal and/or epidural anesthesia.

**Table 1:** **Severity score checklist for symptoms of transurethral resection syndrome**

Severity score	1	2	3
Circulatory			
Chest pain	Duration < 5 min	Duration > 5 min	Repeated attacks
Bradycardia	HR decrease 10–20 bpm	HR decrease > 20 bpm	Repeated decreases
Hypertension	SAP up 10–20 mmHg	SAP up > 30 mmHg	Score (2) for 15 min
Hypotension	SAP down 30–50 mmHg	SAP down > 50 mmHg	Repeated drops > 50 mmHg
Poor urine output	Diuretics needed	Repeated use	Diuretics ineffective
Neurological			
Blurred vision	Duration < 10 min	Duration > 10 min	Transient blindness
Nausea	Duration < 5 min	Duration 5–120 min	Intense or > 120 min
Vomiting	Single instance	Repeatedly, < 60 min	Repeatedly, > 60 min
Uneasiness	Slight	Moderate	Intense
Confusion	Duration < 5 min	Duration 5–60 min	Duration > 60 min
Tiredness	Patient says so	Objectively exhausted	Exhausted for > 120 min
Consciousness	Mildly depressed	Somnolent < 60 min	Needs ventilator
Headache	Mild	Severe < 60 min	Severe > 60 min

A checklist used to define and score the clinical manifestations of transurethral resection syndrome [6].

HR, heart rate; SAP, systolic arterial pressure.

Venous blood samples were obtained at least once intraoperatively, as well as 3 h after the end of surgery. If any neurological manifestations were observed, blood gas analysis was performed several times to treat hyponatremia, thus avoiding further deterioration.

Neurological score was defined as the total number of neurological manifestations, including restlessness, nausea, vomiting, pain, and confusion. The correlation between serum sodium levels and neurological scores was investigated.

Patients were divided into two groups: those with and without clinical manifestations of TUR syndrome. Factors compared in these two groups included patients’ demographic characteristics, weight of the resected prostate, operating time, infusion and transfusion volumes, manifestations of TUR syndrome, postoperative blood test results, and whether the irrigation fluid was or was not continuously drained through a suprapubic pigtail drainage catheter (C. R. Bard, Inc., Karlsruhe, Germany) [7].

Parameters in patients with and without symptoms of TUR syndrome were compared using Mann–Whitney U tests, unpaired t-tests, Fisher’s exact tests, and chi-squared tests, as appropriate. Spearman’s correlation coefficient was calculated to evaluate the relationship between neurological scores (defined as the total number of neurological manifestations, including restlessness, nausea, vomiting, pain, and confusion) and changes in serum sodium levels. Receiver operator characteristic (ROC) curve analysis was performed to determine the predictive value and optimal cutoff point of change in serum sodium level for the clinical manifestations of TUR syndrome. A p value of < 0.05 was considered statistically significant. All statistical analyses were performed using GraphPad Prism version 5.0 for Mac (GraphPad Software, San Diego, CA, USA). Univariate logistic regression analyses were performed for parameters judged to be risk factors for TUR syndrome in the literature [7], including age, body weight, and operating time, and so on. Only observational variables with p < 0.05 in univariate analyses were included in the logistic regression model to ascertain their independent effects on the symptom occurring. The odds ratio and p values were calculated for each variable. Again a p value of < 0.05 was considered statistically significant. The logistic regression analyses were performed with SPSS version 22.0 for Mac (SPSS, Chicago, IL, USA).

## Results

Of the 229 patients included in this study, 60 (26.2%) developed clinical manifestations of TUR syndrome and 169 (73.8%) did not. Most cardiovascular abnormalities were hypertension with reflex bradycardia, or sudden hypotension, which is in agreement with our previous findings [8]. Baseline demographic and clinical characteristics were similar in symptomatic and asymptomatic patients, except for preoperatively estimated prostatic grand size (Table 2). However, the two groups showed significant differences in the duration of surgery, weight of resected specimen, volumes of intravenous infused liquid and transfused blood volume, continuous drainage of irrigation fluid via suprapubic cystostomy, minimum serum sodium concentrations, and postoperative blood sampling (Table 3). Minimum serum sodium level means the smallest amount of sodium detected in each patient in the intraoperative and postoperative period. Eight patients required sedatives, diuretic drugs, and/or sodium to alleviate these symptoms and correct their hyponatremia. Patients with severe symptoms such as confusion required additional intravenous anesthetic agents with appropriate airway management. In addition, of the 195 patients who maintained a normal sodium level in the intra- and post-operative period, 31 patients showed symptoms. Of the 34 patients with sodium level less than or equal 125 mmol/l, five patients did not show any symptoms.

**Table 2:** **Baseline demographic and clinical characteristics of patients that underwent transurethral resection of the prostate**

Parameter	Symptomatic (n = 60)	Asymptomatic (n = 169)	*P*value
Age, yr	72 ± 8	70 ± 7	0.146
Height, cm	164.6 ± 6.2	164.8 ± 6.0	0.788
Body weight, kg	62.9 ± 10.0	63.2 ± 9.5	0.862
Diabetes mellitus	4 (66.7%)	11 (6.5%)	1.000
Hypertension	6 (10.0%)	14 (8.3%)	0.395
Arrhythmia	1 (1.7%)	1 (0.6%)	0.456
Preoperative blood data			
Creatinine, mg/dl	0.9 ± 0.2	0.9 ± 0.2	0.861
BUN, mg/dl	16.6 ± 6.1	15.7 ± 4.1	0.619
Sodium, mmol/l	140.8 ± 2.1	140.7 ± 2.3	0.963
Hemoglobin, g/dl	13.6 ± 1.5	14.0 ± 1.5	0.078
Estimated prostatic grand size, g	87.8 ± 41.8	64.9 ± 26.6	<0.001

Data expressed as mean ± SD or number (%). Transurethral resection syndrome was defined as the presence of central nervous system disturbances such as nausea, vomiting, restlessness, pain, confusion, or even coma with circulatory abnormalities both intra- and post-operatively.

Asymptomatic, patients with no signs of transurethral resection syndrome; Symptomatic, patients with signs of transurethral resection syndrome; BUN, blood urea nitrogen.

**Table 3:** **Operative and postoperative data for patients that underwent transurethral resection of the prostate**

Parameter	Symptomatic (n = 60)	Asymptomatic (n = 169)	*P*value
Continuous irrigation fluid drainage	23 (38.3%)	16 (9.5%)	<0.001
0.5% Bupivacaine, ml	2.5 ± 0.4	2.5 ± 0.3	0.117
Resection weight, g	47.5 ± 29.6	31.1 ± 20.0	<0.001
Operation time, min	108 ± 35	85 ± 35	<0.001
Infusion volume, ml	909 ± 508	635 ± 346	<0.001
Infusion and transfusion volume, ml	1107 ± 609	697 ± 385	<0.001
Diuretics	7 (11.7%)	1 (0.6%)	<0.001
Sodium chloride	1 (1.7%)	0 (0.0%)	0.143
Symptoms	60 (100.0%)	-	NA
Restlessness	28 (46.7%)	-	NA
Vomiting	22 (36.7%)	-	NA
Nausea	36 (60.0%)	-	NA
Pain	17 (28.3%)	-	NA
Confusion	13 (21.7%)	-	NA
Postoperative blood data			
Creatinine, mg/dl	0.9 ± 0.3	0.9 ± 0.2	0.774
BUN, mg/dl	12.9 ± 5.2	13.0 ± 4.0	0.368
Sodium, mmol/l	134.8 ± 6.8	138.2 ± 3.4	<0.001
Hemoglobin, g/dl	11.0 ± 1.8	12.7 ± 1.6	<0.001
Operative and postoperative blood data			
Minimum hemoglobin, g/dl	10.8 ± 2.0	12.4 ± 1.9	<0.001
Minimum sodium, mmol/l	125.2 ± 10.5	136.9 ± 4.5	<0.001
Minimum sodium ≤125 mmol/l	29 (48.3%)	5 (3.0%)	<0.001
Change in sodium level, mmol/l	14.9 ± 10.3	3.8 ± 4.5	<0.001
Percent change in sodium level, %	10.6 ± 7.3	2.7 ± 3.2	< 0.001

Data expressed as mean ± SD or number (%). Transurethral resection syndrome was defined as the presence of central nervous system disturbances, such as nausea, vomiting, restlessness, pain, confusion, or even coma, with circulatory abnormalities both intra- and post-operatively.

NA, not applicable; BUN, blood urea nitrogen.

**Figure 1:**
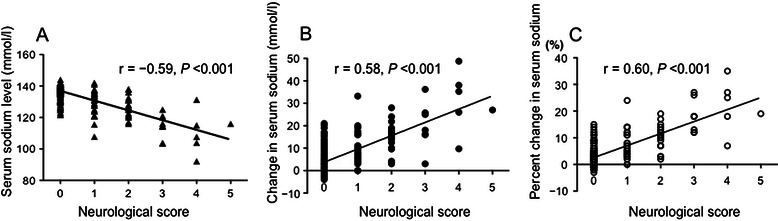
Correlations between neurological scores and serum sodium levels. Clinical neurological manifestations showed (**A**) an inverse correlation with minimum sodium level (Spearman’s correlation coefficient −0.59), and positive correlations with (**B**) changes in absolute sodium levels (Spearman’s correlation coefficient 0.58) and (**C**) percent changes in serum sodium levels (Spearman’s correlation coefficient 0.60). All p values were less than 0.001.

Serum sodium levels in the symptomatic and asymptomatic groups were reduced by 14.9 ± 10.3 and 3.8 ± 4.5 mmol/l, respectively. These values were the absolute values in each patient and they were calculated as (preoperative sodium level) – (minimum sodium level). Percent change was calculated as (absolute value)/(preoperative sodium level). The change in serum sodium levels correlated with clinical neurological manifestations. Neurological manifestations were inversely correlated with minimum sodium concentration (Spearman’s correlation coefficient −0.59), and positively correlated with changes in absolute change of serum sodium concentrations (Spearman’s correlation coefficient 0.58) and percent change of serum sodium concentrations (Spearman’s correlation coefficient 0.60) (Figure 1).

The logistic regression results are shown in Table 4. We made three sets of models to detect the best predictive variable between absolute sodium level, change in sodium level, and percent change in sodium level. The risk factors for symptoms occurring were sodium level variables, operation time longer than 90 min, and presence of continuous drainage from bladder.

**Table 4:** **Results of logistic regression analyses of risk factors for transurethral resection syndrome**

Parameter	P value	Odds ratio	95% Conficdence interval	Hosmer-Lemeshow test	
				P value	Accuracy
Set1				0.168	86.0%
Resected weight≧46 g	0.944	1.03	0.41 to 2.59		
Operating time≧90 min	0.006	3.31	1.40 to 7.82		
Minimum sodium ≦125 mmol/l	<0.001	34.66	11.77 to 102.06		
Continuous irrigation fluid drainage	<0.001	9.62	3.72 to 24.89		
Set2				0.044	81.1%
Resected weight≧46 g	0.664	0.82	0.33 to 2.04		
Operating time≧90 min	0.024	2.61	1.13 to 6.03		
Change in sodium level ≧7 mmol/l	<0.001	14.76	6.66 to 32.72		
Continuous irrigation fluid drainage	<0.001	6.08	2.29 to 16.15		
Set3				0.042	81.6%
Resected weight≧46 g	0.968	0.98	0.40 to 2.41		
Operating time≧90 min	0.015	2.77	1.22 to 6.29		
Percent change in sodium level≧7%	<0.001	15.36	6.82 to 34.58		
Continuous irrigation fluid drainage	<0.001	6.90	2.65 to 17.92		

**Figure 2:**
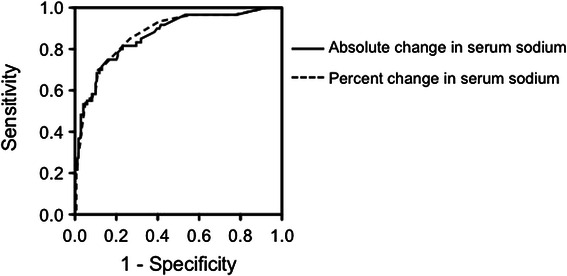
Receiver operating characteristic (ROC) curves showing the ability of change in serum sodium level to predict clinical manifestations of transurethral resection syndrome. ROC analysis showed that the optimal cutoff value for change in serum sodium concentration was 7.4 mmol/l, and that the optimal cutoff value for percent change was 7.0%. All the numerical information are given in Table 5.

ROC analysis showed that the optimal cutoff value for change in absolute serum sodium concentration was 7.4 mmol/l, with an area under the ROC curve (AUC) of 0.87, a sensitivity of 0.72, and a specificity of 0.87 (Figure 2, Table 5). The optimal cutoff value for percent change in serum sodium level was 7.0%, with an AUC of 0.87, a sensitivity of 0.70, and a specificity of 0.89 (Figure 2, Table 5).

**Table 5:** **Results of receiver operating characteristics (ROC) analysis for absolute and percent change in serum sodium levels**

	Cutoff value	AUC	Sensitivity	Specificity	*P*value
Change in serum sodium	7.4 mmol/l	0.87	0.72	0.87	< 0.001
Percent change in serum sodium	7.0%	0.87	0.70	0.89	< 0.001

AUC, area under the ROC curve.

## Discussion

This study defined clinical manifestations of TUR syndrome based on the appearance of cardiovascular and neurological manifestations, and assessed these symptoms relative to changes in serum sodium level [7,8]. Because of the difficulty in scoring cardiovascular manifestations of TUR syndrome, a neurological score system that could indicate the symptomatic severity of hyponatremia was used.

Our ROC analyses found that decreases in serum sodium levels > 7.4 mmol/l and > 7.0% were predictive of the clinical manifestation of TUR syndrome. As normal serum sodium concentrations are about 135–145 mmol/l, clinical manifestations may occur even in patients with serum sodium levels > 125 mmol/l; this indicates that symptoms of TUR syndrome may develop even when serum sodium levels remain within a normal range. In addition, although the outcomes of TURP under general and local anesthesia were previously reported to be similar [9], patients who undergo TURP under general anesthesia may more readily develop dilutional hyponatremia owing to the slow absorption of irrigation fluid into blood vessels [10,11]. Therefore, a long-acting diuretic able to maintain serum sodium levels, such as tolvaptan, may be optimal for preventing TUR syndrome.

Logistic regression analysis identified the risk factors of symptoms occurring as serum sodium level variables, operation time more than or equal to 90 min, and presence of continuous drainage from bladder. We evaluated three predictive variables of serum sodium concentration. A sodium level less than or equal to 125 mmol/l had the highest accuracy, therefore, it was the most appropriate predictive parameter. Change in sodium level and percent change in sodium level had similar predictive values.

In this study, we detected several neurological manifestations of TUR syndrome, including restlessness, nausea, vomiting, pain, and confusion. Visual changes, seizures, and encephalopathy caused by cerebral edema have been previously reported [5], but were not observed in this study. Pain was the most difficult symptom to categorize as being caused by hyponatremia, as it may be caused by the surgery itself. Patients reported vague abdominal pains, accompanied by restlessness or nausea.

Postoperative serum sodium levels were examined 3 h after the end of surgery. None of the patients received postoperative diuretics or sodium chloride. Postoperative serum sodium levels recovered relatively easily. As irrigation fluid contains no electrolytes, the absorption of this fluid into the blood from the bladder can lead to hyponatremia. Water mainly filters into urine automatically in postoperative patients. However, some patients experienced reductions in sodium levels due to abnormal volume shift. Our previous study found that continuous drainage of irrigation fluid through a suprapubic cystostomy was an important risk factor for TUR syndrome in older patients [7]. Although this continuous drainage was designed to remove debris, blood, and clots from the operating field, it may accelerate dilutional hyponatremia. In the patients’ backgrounds, there was a significant difference between the two groups in preoperatively-estimated prostate gland size. Prostate gland size was preoperatively estimated using ultrasonography, which showed that the patients in the symptomatic group had larger prostates than those in the asymptomatic group; this is in agreement with our previous findings [8].

The major limitation of this study was the inability to obtain frequent blood samples, as blood was not continuously sampled during surgery. In addition, clinical manifestations may have differed in anesthetic and nursing charts, because the detection of restlessness and nausea is subjective. Cardiovascular manifestations and pulmonary compromise required aggressive interventions such as converting from local to general anesthesia, which may explain our lack of severe neurological scores.

## Conclusion

Decreases in serum sodium levels of > 7.4 mmol/l or > 7.0% could predict the development of cardiovascular and neurological manifestations of TUR syndrome.
